# Better retention of Malaysian opiate dependents treated with high dose methadone in methadone maintenance therapy

**DOI:** 10.1186/1477-7517-7-30

**Published:** 2010-12-17

**Authors:** Nasir Mohamad, Nor Hidayah Abu Bakar, Nurfadhlina Musa, Nazila Talib, Rusli Ismail

**Affiliations:** 1 Pharmacogenetic Research Group, Institute for Research in Molecular Medicine (INFORMM), Health Campus, Universiti Sains Malaysia, 16150 Kubang Kerian, Kelantan, Malaysia; 2 Department of Emergency Medicine, School of Medical Sciences, Health Campus, Universiti Sains Malaysia, 16150 Kubang Kerian, Kelantan, Malaysia

## Abstract

**Background:**

Methadone is a synthetic opiate mu receptor agonist that is widely used to substitute for illicit opiates in the management of opiate dependence. It helps prevent opiate users from injecting and sharing needles which are vehicles for the spread of HIV and other blood borne viruses. This study has the objective of determining the utility of daily methadone dose to predict retention rates and re-injecting behaviour among opiate dependents.

**Methods:**

Subjects comprised opiate dependent individuals who met study criteria. They took methadone based on the Malaysian guidelines and were monitored according to the study protocols. At six months, data was collected for analyses. The sensitivity and specificity daily methadone doses to predict retention rates and re-injecting behaviour were evaluated.

**Results:**

Sixty-four patients volunteered to participate but only 35 (54.69%) remained active and 29 (45.31%) were inactive at 6 months of treatment. Higher doses were significantly correlated with retention rate (p < 0.0001) and re-injecting behaviour (p < 0.001). Of those retained, 80.0% were on 80 mg or more methadone per day doses with 20.0% on receiving 40 mg -79 mg.

**Conclusions:**

We concluded that a daily dose of at least 40 mg was required to retain patients in treatment and to prevent re-injecting behaviour. A dose of at least 80 mg per day was associated with best results.

## Background

Opioid dependence and injecting drug use is a serious world-wide problem. As the global epidemic of heroin use continues, it adds an increasing burden, driving the AIDS epidemic in Malaysia and other parts of Asia, with consequent additional health, economics and social problems. The primary modes of transmission of HIV remain to be unprotected penetrative sex and injection-drug use although other modes also contribute. Direct blood contact as in the sharing of drug-injection equipment, is a particularly efficient means of transmitting the virus. In parts of South East Asia and in Malaysia, the epidemic is driven by injection-drug use. In Malaysia it is opiate drug use. Malaysia has had to grapple with drug use problems for as long as it can be remembered. Despite the avowed objective of becoming "drug-free" by 2015, the country is still struggling to rid itself of the menace. In 1986, HIV landed in Malaysia and soon it got into the drug user population in the country and injecting opiate use is now feeding the Malaysian epidemic [1]. Thus, of the 80,938 cumulative number of HIV infection in the country at the end of 2007, 58,135 were injecting drug users [2]. The management of opiate dependence thus presents a great challenge.

Methadone is a μ-opiate receptor agonist developed by German scientists in the late 1930s. It was approved by the U.S Food and Drug Administration (FDA) in 1947 as a painkiller, and by 1950 oral methadone also was used to treat the painful symptoms of persons withdrawing from heroin [1,3,4]. In 1964, Dole's team discovered that continous daily doses of oral methadone were beneficial, allowing otherwise debilitated opioid addicts to function more normally [5-11]. An adequate maintenance dose of methadone does not make the patient feel "high' or drowsy, so the patient can generally carry on a normal life. If used properly, methadone is generally safe and non-toxic with minimal side effect [5-11].

With the advent of HIV/AIDS in the 80's it slowly assumed a central role in the management of opiate dependence where it has probably revolutionized the approach with the adoption of maintenance therapy. Methadone maintenance reduces injection drug use [12] and is effective in reducing illicit heroin use, HIV risk behaviors, HIV and other harms associated with illicit opiate/heroin use [12-14]. Methadone prevents abstinence syndrome, reduces narcotic cravings and block the euphoric effects of illicit opiate use while reducing the risk for HIV and other BBV transmission. The ability of methadone to stabilize opiate dependent individuals provides a platform for addressing the other biological and social dimensions of opiate dependence. Many countries have adopted MMT and MMT is now the most widely used and effective pharmacologic treatment for opiate dependence [15,16]. Apart from bringing health benefits, MMT also reduces illicit opiate use, improves personal and social functioning and reduces drug related crimes [1].

Harm reduction came to Malaysia via the Harm Reduction Working Group at the Malaysia AIDS Council. Their efforts culminated in the Government approving harm reduction in 2003 but it was not until 2005-2006 that Methadone Maintenance Therapy (MMT) was introduced to be followed by the Needle and Syringe Exchange Program (NSEP) and the provision of condoms [17] apart from the provision of the usual educational material. As it is with many diseases, MMT is not a cure-all. It is for this that NSEP is offered. In the private sector in Malaysia, patients may also obtain alternative therapy with buprinorphine.

Methadone maintenance therapy (MMT) is generally considered as corrective therapy, rather than as a "cure" for opioid addiction, and it had no or only limited efficacy in treating dependence on other substances of abuse [1]. At appropriate doses it not hinder a patient's intellectual capacities or abilities to perform tasks [18] and it can correct the compulsive use of heroin and other opiates by addicts [6].

Many factors contribute to the success of MMT but studies have shown that adequate methadone dosing is critical [19]. This is because the dose used must be able to prevent withdrawal, to block craving and perhaps to also block the effect of additional heroin to discourage patients from reverting to heroin. For therapeutic success, adequate methadone dosing is critical. World-wide, the dose of methadone varies, with a tendency for a relatively low daily dose [20]. Malaysia is no exception. Indeed in private practice in Malaysia, the average dose is only 10-20 mg methadone per day, and only 5% of patients receive doses greater 80 mg/day [21]. Even in the government sector where methadone is provided free, average daily dose is below 40 mg [22]. The reason for this is probably not related to the variable pharmacology of methadone but rather because many physicians, have at the back of their mind, a belief that "zero drug is best". Some physicians also fear the severe adverse drug response and fatal cardiac problems with methadone [23,24]. Inadequate doses and premature termination are the greatest threats to a successful MMT program in Malaysia. Malaysian doctors tend to use low doses despite the fact that the traditional dosing with lower doses are expected to be ineffective [25]. Malaysian doctors may outwardly say that they use lower methadone doses because of their fear for ethnic difference that would put their patients at higher risks for toxicity if they were to use doses as high as those recommended by the Western literature. What they may not want to admit is the fact that, inwardly, they have fears with methadone (and all opiates actually!) just for the simple reason that methadone is an opiate, just like the dreaded heroin and morphine!. Indeed Malaysian doctors are not alone in this. Many doctors everywhere share the same view. Thus, despite ample evidence for the need to maintain patients at a daily dose of 80 mg to 100 mg, most patients are maintained on much less, and many are encouraged early termination. It is probably understandable that the lay public may not understand the scientific basis for MMT and could be disparaging and become critical of it. It is however less clear why many physicians and other health care providers have the same views. Even those directly involved with MMT programs frequently fail to adhere to the basic principles of MMT. Most have actually received clear information on the pharmacologic principles underlying MMT and their claim that they want to prescribe as few medications as possible sound hollow, as they frequently easily prescribe other mood altering drugs, such as the benzodiazepines that are often prescribed with abandon and can produce psychological and physiologic dependency. Even if they claim they fear adverse effects, the adverse, physiologic effects of MMT are minimal and methadone is probably associated with the least side effects of any drug in a physician's pharmacologic armamentarium, when used appropriately. The real reason is probably more to do with the general "opiophobias" as it is known that some doctors even hesitate to use opiates even when indications are clear. Efforts should therefore be made urgently to reeducate these doctors. In their hands is the future of the nation. Their failure to prescribe adequate methadone doses will lead to therapeutic failure for MMT. This has dire consequences.

Our physicians justify the lower methadone doses used on account of the smaller body sizes of their [26] and the possibility of reduced drug metabolisms [27]. However, drug metabolism and drug doses do not depend on the body weight alone. It is more likely to be related to the expression level and catalytic activity of the putative drug metabolising enzymes (DME) in the individual patients [28]. With methadone, metabolism is complex, mediated by several polymorphic DMEs as reflected by the large variability observed with methadone disposition and half lives [29-31]. DME polymorphisms have large geographic and ethnic variations [32]. With CYP2B6 and CYP3A4, two enzymes that have been implicated in methadone metabolism, the ethnic groups in Malaysia show polymorphism with types and frequencies that differed from each other and from ethnic groups in other geographic location [33]. Furthermore, the frequencies for mutations at CYP3A4 locus were found to be higher among Malay opiate dependent individuals compared to non-opiate dependent Malays. CYP3A4 is an enzyme whose activity is also altered by environmental factors like char-broiled food and grape fruits [34]. All these would probably have more impact on methadone dose requirements in Malaysia than would body weights. However, given the body of the literature that support the need for adequate dose, the low doses used can lead to reduced effectiveness and thus negating the objective of the programmed.

Remaining in treatment for an adequate period of time is critical for treatment effectiveness. The appropriate duration for an individual depends on their problems and needs, but research indicates that for most drug users, the threshold of significant improvement is reached only after about three months in treatment, with further gains as treatment is continued. Because people often leave treatment prematurely, and premature departure is associated with high rates of relapse to drug use, programmes need strategies to engage and keep patients in treatment.

This paper reports retention rates and injecting behaviour in pilot patients given different doses of daily methadone in MMT programme, two parameters that are very important for MMT in preventing the spread of blood borne viruses. This study was performed to provide a platform for further studies on the pharmacologic optimisation of methadone dose to attain its maximum benefits.

## Methods

### Patients

The study was approved by the ethical committee at the University of Malaya Medical Centre. Patients comprised opiate dependent individuals who met the inclusion and exclusion criteria (Table 1) of the national MMT program. They gave a written consent prior to the enrollment. They were initiated on a methadone dose based on the guidelines of the Malaysian Ministry of Health. The administration of methadone was directly observed by the prescribing physician. Patients were monitored according our study protocols and the guidelines to monitor opiate usage and injecting behavior. At six months, data was collected for analyses.

**Table 1:** The criteria of enrollment as recommended by Ministry of Health, Malaysia

Criteria	Inclusion	Exclusion
	1. Opiate dependence by DSM IV criteria	1. Defaulter treatment for more than 3 days.
	2. Age 18 years and above	2. Behavior judged by treating physician to be destructive to MMT clinic.
	3. Willing to comply with the daily Direct Observation Therapy Supervision (DOTS) and dosing.	

### Statistical Methods

At the end of six months, patients' data were collated. Patients were divided into 3 groups according to the daily dose level that they received, "low dose" (0-39 mg), "intermediate dose" (40-79 mg) and "high dose" (80 mg or more) [19]. Summary statistics were calculated.

Differences between groups were tested for significance using the chi-square test and p value of less than 0.05 was taken as signifying statistical significance.

Additionally, sensitivity, specificity, positive predictive value (PPV) and negative predictive value (NPV) were also calculated using the following formula and definition. This was done with the view of using daily dose groups to predict successful methadone therapy, i.e., knowing the daily dose, how sure we are that MMT will give good outcomes in the affected patients. For the purpose of data analysis "inadequate dose" methadone group and "adequate dose" methadone groups were classified. "inadequate dose" group is defined as daily methadone doses of less 80 mg and "adequate dose" group is defined as daily methadone dose of more than 80 mg.

$$\text{Sensitivity}=\frac{\text{Number of true positive}}{\text{Number of true positive}+\text{Number of false negative}}\times 100\%$$

$$\text{Specificity}=\frac{\text{Number of true negative}}{\text{Number of true negative}+\text{Number of false positive}}\times 100\%$$

$$\text{PPV}=\frac{\text{Number of true positive}}{\text{Number of true positive}+\text{Number of false positive}}\times 100\%$$

$$\text{NPV}=\frac{\text{Number of true negative}}{\text{Number of true negative}+\text{Number of false negative}}\times 100\%$$

For retention rate, the true positive is defined as those patients on "adequate dose" who remain active on treatment, true negative is defined as those patients on "inadequate dose" and are defaulter to treatment, false positive is defined as those patients on "inadequate dose" but remain active and false negative is defined as those patients on adequate dose but defaulted treatment.

For re-injecting behavior, the true positive is defined as those patients on "adequate dose" and not re-injecting, true negative is defined as those patients on "inadequate dose" and has re-injecting behavior, false positive is defined as those patients on "inadequate dose" but show no re-injecting behavior and false negative is defined as those patients on "adequate dose" but exhibit re-injecting behavior. All statistics were performed using SPSS (SPSS Inc, Ill, v 13) on an IBM-compatible computer.

## Results

Sixty-four patients were enrolled for this pilot study. All were Malay males. The youngest was 20 years old and the oldest 56. All have had a long history of illicit drug use exceeding 2 years. The youngest age at which they first took illicit drugs was 12 years. Their doses were titrated appropriately as tolerated and the final dose averaged 57.2 mg (SD ± 22.7) with a range from 20 to 160 mg per day and a median of 50 mg. Table 2 details the characteristics of the study patients.

**Table 2:** Demography of study patients

Details	N	%
Gender:		
-Male	64	100
-Female	0	
Age:		
-Youngest	20	
-Eldest	56	
-Mean:	33	
Age at 1^st ^time illicit drug use		
-As young as	12 year-old	
-As old as	32 year-old	
-Mean	20 year-old	
Duration of Opiate Addiction:		
-Minimum	2 years	
-Maximum	38 years	
-Mean	13 years	
HIV infection status		
-Positive	23	36
-Negative	37	58
-Unknown	4	8

Overall retention rate at 6-month was 54.69% with 29 patients lost to follow up. Of the "retained" patients, 80% were receiving doses of 80 mg or more per day. None was found in the 0 - 39 mg per day dose group. Retention rate for the 40 - 79 mg per day dose group was 20%. As shown in Table 3 and 4, the differences between the dose groups in terms of retention rates reached and re-injecting behavior statistical significance (p = 0.001).

**Table 3:** Retention status of patients in MMT programme at 6 months follow-up

Retention Status at 6 months		Group of Methadone Doses		Total	p-value	Sensitivity	Specificity	Positive predictive value (PPV)	Negative predictive value (NPV)
								
		80 mg & below	80 mg & above						
Active	n	7	28	35					
	%	20	80	100					
Defaulter	n	27	2	29	0.001	80.00%	93.10%	93.30%	79.40%
	%	93	7	100					
Total	n	34	30	64					
	%	53	47	100					

**Table 4:** Re-injecting behaviour of patients in MMT programme at 6 months follow-up

Re-injecting behaviour at 6 month		Group of Methadone Doses		Total	p-value	Sensitivity	Specificity	Positive predictive value (PPV)	Negative predictive value (NPV)
								
		80 mg & below	80 mg & more						
Yes	n	24	9	33	0.001	71.00%	73.00%	71.00%	73.00%
	%	73	27	100					
No	n	9	22	31					
	%	29	71	100					
Total	n	33	31	64					
	%	52	48	100					

In terms of using methadone daily doses greater than 80 mg as a predictor of a successful outcome for MMT (as measured by retention rates and re-injection behavior), we found that the positive predictive value of doses greater than 80 mg a day to predict retention was 80% and its negative predictive value was 93%. This means that, in terms of predicting retention, a daily dose exceeding 80 mg will have a probability of 0.8 in accurately predicting that the patient will be retained in treatment. In terms of predicting failure to retain, it has a probability of 0.93 in making an accurate prediction. A similar probability was also calculated with regards re-injecting behavior but in terms of predicting non-re-injecting, the probability of accurate prediction is only 0.73.

## Discussion

Over time, many important discoveries have revolutionized the practice of medicine. The discovery of penicillin and other antibiotics for instance, have changed the ways infectious diseases are treated and the discovery of X-rays has introduced new ways for diagnostics. Methadone could have occupied a similar position but for the stigma and discrimination that drug use disorder and opiate use suffer from.

The 21^st ^century saw Malaysia leading the pact of countries with a most rapidly rising HIV epidemic. After tireless efforts by individuals and organizations, MMT was finally instituted in the country. The program is now implemented at public and private health facilities and other facilities involved with drug use communities. The primary goal is to blunt the rapid rise in HIV infections although the public mainly see it as a treatment for drug addiction. This paper reports a finding from a pilot project on MMT in a small cohort of injecting drug users in SAHABAT and Klinik Dr Khafiz. It is intended to provide a basis for a more comprehensive research to understand factors that can contribute to successes with MMT as, among heroin drug users, MMT has demonstrated effectiveness in reducing HIV risk behaviors and HIV infection [12-14]. MMT programs in Malaysia are implemented in government hospitals as well as in private practice.

SAHABAT is a non-governmental organisation working for and with drug use communities in Kelantan. MMT was introduced in SAHABAT in 2008. SAHABAT is the first centre in Malaysia to have both the MMT program and NSEP running under one roof. SAHABAT also boasts as the only NGO-run centre that was allowed to prescribe methadone. The other centre included in this study was Klinik Dr Khafiz, a general practice clinic near Kuala Lumpur. It was included to provide an insight of MMT practice in the community.

In this study, all the patients enrolled were males in the productive age group. This underscores the importance of proper management of opiate addiction because of its potential influence on population growth, demography as well as productivity of the nation as young males play a significant role in providing Malaysia's work force. Of note was a high prevalence of HIV positivity. In most countries that practice harm reduction among injecting drug users, the incidence of HIV positivity is generally 1-2% [35]. The high prevalence seen in our Malaysian cohort suggests the need for urgent effective measure to control. This is now done in Malaysia with MMT and NSEP.

Drug use disorder is a chronic relapsing disease. Our study revealed that no age group is spared. Our youngest patient was 20 years-old. They began their drug habit as early as when they were 12 years. The oldest patient was 56 years and the oldest age a patient started with the habit was 32 years. The duration of illness among our patients ranged from two years to 38 years and averaged 13 years. These have implications. For one, preventive measures for drug use disorder must begin early and should be continued through all ages. Patients afflicted with the disease should also have long follow ups as they evidently continue with their habits right through their golden years. The longer they continue on the habit, the greater is the chance for them to contract diseases like HIV, if they have not yet been infected. Being young and otherwise healthy, young addicts may find themselves constrained in various activities and this may lead them to many other unhealthy practices.

Drug users do not live in isolation. Apart from transmission through the sharing of injection equipments, having the HIV reservoir, drug users can also transmit the disease to their sexual partners, through penetrative sex. Thus, what started in Malaysia as a concentrated epidemic among drug users is now showing evidence for a more generalized epidemic through sexual transmission. In the beginning, less than one percent of HIV victims were females. Now it stands at about 20% and this clearly demonstrates the generalization of the HIV epidemic in Malaysia. Most of the afflicted females are also wives and spouses of drug users who are themselves HIV positive and not sex workers as many would have expected. There is however evidence for a growing epidemic among sex workers and this again has the potential to generalize into the community.

A most important characteristic of a good MMT program is high retention rate [36]. Our overall retention rate at 6 months was low. Coupled with the relatively small percentage of opiate-dependent individuals having access to MMT in Malaysia, this may threaten the success of MMT as a tool to reduce HIV spread in Malaysia. The low retention rate we saw was probably due to the low daily maintenance dose of methadone our patients got. Our daily doses averaged 57 mg. Its median was lower at 50 mg. Our results revealed that best retention rates were obtained among patients treated with 80 mg or more methadone per day. In parallel, our patients treated with 80 mg or more methadone per day showed the least tendency for re-injecting. It is therefore interesting to note that, despite claims by many physicians that relatively lower doses of methadone would be sufficient for our Malaysian patients, our results showed otherwise.

Our findings were also in parallel with studies that showed a sufficiently high dose was required for improved outcomes [37]. High doses suppress illicit heroin use and improve retention and outcomes [38,39]. Dole's original research discovered that 80 to 120 milligrams of methadone per day, on average, was an effective dose. Dozens of studies since then have demonstrated that dosing in that range resulted in superior treatment outcomes, such as better retention of patients in treatment and less illicit drug use [9,11,39]. Patients maintained on inadequately low doses are much more likely to use illicit opioids and respond poorly to therapy [18]. A study by Strain et al [40] also concluded that patients receiving 80 mg or more methadone per day had significantly greater decreases in illicit opiod use. Another study concluded that a sufficiently high dose of substitution therapy was required for improved outcome [37] and many other independent studies also showed that high doses of methadone were significantly more effective in suppressing illicit heroin use and in retaining patients in the treatment [38,41,42].

Inadequate doses and premature termination will probably be the greatest threats to a successful MMT program in Malaysia. Malaysian doctors tend to use low doses despite the fact that the traditional dosing with lower doses are expected to be ineffective [43]. Many also actively encourage their patients to terminate MMT early. They may outwardly say that they use lower methadone doses because of fear for ethnic difference that would put patients at risks for toxicity. They will not admit their fears with methadone (and all opiates actually!) just for the simple reason that methadone is an opiate, just like heroin and morphine!. Malaysian doctors are not alone in this. Despite evidence for the need for a daily dose of 80 mg to 100 mg, most patients are maintained on much less. It is probably understandable that the lay public may not understand the scientific basis for MMT and could be disparaging and become critical of it. It is however less clear why many physicians and other health care providers have the same views. Most have actually received clear information on the principles underlying MMT. They may also claim that they want to prescribe as few medications as possible but this sounds hollow. Many frequently easily prescribe other mood altering drugs, such as the benzodiazepines that can also produce psychologic and physiologic dependency. Even if they claim they fear adverse effects, the adverse, physiologic effects of MMT are minimal and methadone is probably associated with the least side effects of any drug in a physician's pharmacologic armamentarium, when used appropriately. Illicit use of benzodiazepines, even among MMT patients, are now threatening to derail the MMT program as many continue to inject benzodiazepines although they may have discontinued injecting opiates. There is probably a general "opiophobias". It is known that some doctors even hesitate to use opiates even when indications are clear. Efforts should therefore be made urgently to reeducate these doctors. In their hands is the future of the nation. Their failure to prescribe adequate methadone doses will lead to therapeutic failure for MMT and this has dire consequences.

Our physicians justify the lower methadone doses used on account of the smaller body sizes of their [26] and the possibility of reduced drug metabolisms [27]. However, drug metabolism and drug doses do not depend on the body weight alone. It is more likely to be related to the expression level and catalytic activity of the putative drug metabolising enzymes (DME) in the individual patients [28]. With methadone, metabolism is complex, mediated by several polymorphic DMEs as reflected by the large variability observed with methadone disposition and half lives [29-31]. DME polymorphisms have large geographic and ethnic variations [32]. With CYP2B6 and CYP3A4, two enzymes that have been implicated in methadone metabolism, the ethnic groups in Malaysia show polymorphism with types and frequencies that differed from each other and from ethnic groups in other geographic location [33]. Furthermore, the frequencies for mutations at CYP3A4 locus were found to be higher among Malay opiate dependent individuals compared to non-opiate dependent Malays. CYP3A4 is an enzyme whose activity is also altered by environmental factors like char-broiled food and grape fruits [34]. All these would probably have more impact on methadone dose requirements in Malaysia than would body weights.

Nevertheless, as with many drugs, the dosing of methadone must be individualised [19]. Too low a dose will lead to relapse and failure whereas too high a dose will lead to toxicity such as prolongation of QT interval and subsequent fatal polymorphic ventricular fibrillation [20]. For some reasons such as, pharmacologic variability at the enzyme and receptor levels, high tolerance to opioids, physical condition, mental status, concurrent medications, or prior use of high-purity heroin, however, some patients require much higher daily methadone doses for treatment success, sometimes exceeding 200 mg/day or more [10,11,44].

## Conclusions

We conclude that MMT in Malaysia is faced with the challenge of retaining patients in the program as a relatively low retention rate was found in our cohort of patients. For this, an adequate daily dose of methadone (typically at least 80 mg per day) was an important modifiable determinant to achieve higher retention rates and minimizing re-injecting behaviour among these opiate dependent individuals on MMT. Knowledge of daily methadone doses is a useful predictor of a successful MMT.

## Competing interests

The authors declare that they have no competing interests.

## Authors' contributions

**NM **carried out the study design, recruited subject, collecting data, analysing and interpreting the data, and drafted the manuscript. **NHAB **participate in analysing and interpreting the data, and drafted the manuscript. **NMS **involve in collecting data. **NT **involve in collecting data. **RI **carried out the study design, analysing and interpreting the data, and drafted the manuscript. All authors read and approved the final manuscript.
